# Novel Gene-Based Analysis of ASD GWAS: Insight Into the Biological Role of Associated Genes

**DOI:** 10.3389/fgene.2019.00733

**Published:** 2019-08-09

**Authors:** Aitana Alonso-Gonzalez, Manuel Calaza, Cristina Rodriguez-Fontenla, Angel Carracedo

**Affiliations:** ^1^Grupo de Medicina Xenómica, Fundación Instituto de Investigación Sanitaria de Santiago de Compostela (FIDIS), Center for Research in Molecular Medicine and Chronic Diseases (CIMUS), Universidad de Santiago de Compostela, Santiago de Compostela, Spain; ^2^Grupo de Medicina Genómica, CIBERER, CIMUS (Centre for Research in Molecular Medicine and Chronic Diseases), Universidade de Santiago de Compostela, Santiago de Compostela, Spain

**Keywords:** autism spectrum disorder, gene-based analysis, neurodevelopmental disorders, differential expression analysis, gene-network analysis, genome-wide association study, single-nucleotide polymorphism

## Abstract

**Background:** Autism spectrum disorder (ASD) is a neurodevelopmental disorder characterized by its significant social impact and high heritability. The latest meta-analysis of ASD GWAS (*genome-wide association studies*) has revealed the association of several SNPs that were replicated in additional sets of independent samples. However, summary statistics from GWAS can be used to perform a gene-based analysis (GBA). GBA allows to combine all genetic information across the gene to create a single statistic (p-value for each gene). Thus, PASCAL (*Pathway scoring algorithm*), a novel GBA tool, has been applied to the summary statistics from the latest meta-analysis of ASD. GBA approach (testing the gene as a unit) provides an advantage to perform an accurate insight into the biological ASD mechanisms. Therefore, a gene-network analysis and an enrichment analysis for KEGG and GO terms were carried out. GENE2FUNC was used to create gene expression heatmaps and to carry out differential expression analysis (DEA) across GTEx v7 tissues and Brainspan data. dbMDEGA was employed to perform a DEG analysis between ASD and brain control samples for the associated genes and interactors.

**Results:** PASCAL has identified the following loci associated with ASD: *XRN2*, *NKX2-4*, *PLK1S1*, *KCNN2*, *NKX2-2*, *CRHR1-IT1*, *C8orf74* and *LOC644172*. While some of these genes were previously reported by MAGMA (*XRN2*, *PLK1S1*, and *KCNN2*), PASCAL has been useful to highlight additional genes. The biological characterization of the ASD-associated genes and their interactors have demonstrated the association of several GO and KEGG terms. Moreover, DEA analysis has revealed several up- and down-regulated clusters. In addition, many of the ASD-associated genes and their interactors have shown association with ASD expression datasets.

**Conclusions:** This study identifies several associations at a gene level in ASD. Most of them were previously reported by MAGMA. This fact proves that PASCAL is an efficient GBA tool to extract additional information from previous GWAS. In addition, this study has characterized for the first time the biological role of the ASD-associated genes across brain regions, neurodevelopmental stages, and ASD gene-expression datasets.

## Introduction

Autism spectrum disorder (ASD) is a neurodevelopmental disorder (NDD) characterized by impaired social interaction and communication together with repetitive and restrictive behaviors (American Psychiatric Association, 2013). The estimated prevalence of ASD stands at approximately 1% in the general population, and this value is influenced by whether adult or child and adolescent populations are considered (The National Academies of Sciences 2015).

ASD is a multifactorial disorder, meaning that several factors (both environmental and genetic) are involved in the development of this condition. Thus, rare genetic variation, including small deletions and duplications (indels), CNVs (*copy number variation*) and SNVs (*single-nucleotide variation*) contribute significantly to ASD liability. In addition, the pattern of inheritance (common or rare) and the origin of these mutations (inherited or *de novo*) will also differentially contribute to the genetic risk. Thus, rare inherited variation (*MAF* < 1%) alone only explains 3% of ASD genetic risk (Sanders et al., 2015). However, common variation accounts for a substantial fraction of ASD heritability (50%) (Genomics et al., 2013; Smoller et al., 2013; Glessner et al., 2014; Anttila et al., 2018). Early GWAS of ASD have failed to detect strong signals which could possibly be due to the presence of phenotypic heterogeneity across collections and the need of much larger sample sizes to achieve an adequate statistical power (Weiss et al., 2009; Anney et al., 2010; Ma et al., 2010). Different associated signals were considered as plausible risk variants for years even when subsequent studies did not replicate most of them. Thus, some examples of associated SNPs (*single-nucleotide polymorphisms*) are the following: rs10513025 (*SEMA5A*), rs4141463 (*MACROD2*), and rs4307059 (*MSNP1*), among others (Weiss et al., 2009; Kerin et al., 2012; Jones et al., 2013; Torrico et al., 2017). However, the genetic landscape of ASD has evolved over the past 10 years, and the latest ASD GWAS meta-analysis conducted by the Psychiatric Genomic Consortium (PGC) is a proof of that. PGC has made an incredible effort not only to increase sample size up to 10,000 cases and controls but also to develop well-defined quality control and imputation pipelines. The latest ASD GWAS meta-analysis has led to the identification of 93 genome-wide significant markers of which only 53 were replicated in independent cohorts. Top associated SNPs were rs910805 (chromosome 20; p-value: 2.04 × 10^−9^) and rs10099100 (chromosome 8; p-value: 1.07 × 10^−8^), among others (Grove et al., 2019). However, GWAS information can be also analyzed following a gene-based analysis (GBA) approach. Thus, p-values for each SNP within a gene are combined to obtain a single statistic for each single gene across the genome (Huang et al., 2011). GBA strategies employ summary statistics from GWAS without the need of individual genotypes. The study of genes as testing units with a biological entity provides the correct framework to carry out secondary approaches. These additional studies help to characterize the molecular and cellular mechanisms involved in the pathogenesis of the disease (Wang et al., 2007).

MAGMA is one of the most commonly used GBA methods together with VEGAS. (de Leeuw et al., 2015; Liu et al., 2010). Thus, the latest ASD GWAS meta-analysis includes an additional GBA done by MAGMA. The top associated genes reported in this study were *KIZ* and *XRN2* (chromosome 20), *MFHAS1*, *XKR6*, *MSRA*, and *SOX7* (chromosome 8). The remaining associated genes were *KCNN2*, *KANSL1*, *MACROD2*, *WNT3*, *MAPT*, *CRHR1*, *NTM*, *MMP12*, and *BLK* (Grove et al., 2019).

PASCAL (*Pathway scoring algorithm*) is one of the most recent GBA approaches. PASCAL generates gene scores by aggregating SNP p-values from GWAS meta-analysis while correcting for LD (*linkage disequilibrium*) using 1000 Genomes data. Thus, PASCAL creates a pairwise SNP by SNP correlation matrix. MAGMA also allows to correct by LD structure but in a different way. It creates a SNP matrix (principal components) pruning away those SNPs presenting small eigenvalues in the matrix (de Leeuw et al., 2015; Lamparter et al., 2016). Moreover, PASCAL is distinguishable from VEGAS and MAGMA by having more statistical power and by being computationally less demanding (Lamparter et al., 2016).

The main aim of this paper is to further mine the summary data from the meta-analysis of ASD by PASCAL. Although it is expected that the results do not vary much from those genes found by MAGMA, additional analysis will be performed to characterize the functional role of the ASD-associated genes. Therefore, gene-network analysis, enrichment analysis, and meta-analysis of DEG (different expressed genes) in ASD brain will be carried out to gain information about their biological function.

## Methods

### GWAS Meta-Analysis

Summary statistics from the latest ASD GWAS meta-analysis were obtained from the public repository available in the PGC website (http://www.med.unc.edu/pgc/results-and-downloads). The following data set was employed: iPSYCH_PGC_ASD_Nov2017.gz (Grove et al., 2019) which includes the meta-analysis of ASD by the Lundbeck Foundation Initiative for Integrative Psychiatric Research (iPSYCH) and the Psychiatric Genomics Consortium (PGC) released in November 2017.

The data set comprises a total of 18,381 cases and 27,969 controls. Additional information including sample size and ancestry can be found in **Table 1**. Additional information about the genotyping and QC methods employed are available at the PGC website.

**Table 1:** Characterization of ASD cohorts included in PGC GWAS meta-analyses.

Study name		Design study	Sample size (cases/controls)	Ancestry	Diagnostic instrument	Total (cases/controls)
iPSYCH		Case/control	13076/22664	European	ICD10 criteria	18381/27969
PGC	UCLA Autism Center of Excellence (ACE)	Trio	391/391	European	ADI-R and/or ADOS	
	Autism Genome Proyect (AGP)	Trio	2272/2272	European	ADI-R and ADOS	
	Autism Genetic Resource Exchange (AGRE)	Trio	974/974	European	ADI-R and ADOS	
	NIMH Repository, the Montreal/Boston Collection (MOMBOS)	Trio	1396/1396	European	ADI-R, Autism Screening questionnaire, ADOS	
	Simons Simplex Collection (SSC)	Trio	2231/2231	European	ADI-R and ADOS	

ICD10, International Classification of Diseases, 10^th^ revision; ADI-R, Autism Diagnostic Interview—Revised; ADOS, Autism Diagnostic Observation Schedule.

### Gene-Based-Analysis

PASCAL scoring algorithm (https://www2.unil.ch/cbg/index.php?title=Pascal) was applied to the summary statistics from ASD meta-analysis. Individual SNPs from GWAS results were first mapped to genes employing a default ±50 kb window around 5′ and 3′UTRs. The default maximum number of SNPs per gene allowed by PASCAL was 3,000. LD information was retrieved from 1000 Genomes European panel. Bonferroni correction sets the significance cutoff at 2.26 × 10^−6^ (0.05/22135 genes).

### Regional Plots

LocusZoom tool (http://locuszoom.org/) was employed to construct regional plots for the regions containing PASCAL associated genes. To this aim, meta-analysis data including marker name, p-values, odds ratio (OR), chromosome position (start-end), and index SNP were specified for the analysis. The source of LD information used to construct the r^2^ correlation matrix between SNPs in these regional plots was retrieved from hg19/1000 Genomes Nov 2014 EUR (European). The rest of the optional controls were used as default.

### Gene-Network Analysis

FunCoup v.4.0 (http://funcoup.sbc.su.se/search/) was employed to expand the list of associated genes (p < 2.26 × 10^−6^) including its interactors. FunCoup database integrates 10 different types of functional couplings among genes that allow to infer functional association networks: protein interaction (PIN), mRNA co-expression (MEX), protein co-expression (PEX), genetic interaction profile similarity (GIN), shared transcription factor binding (TFB), co-miRNA regulation by shared miRNA targeting (MIR), subcellular co-localization (SCL), domain interactions (DOM), phylogenetic profile similarity (PHP), and quantitative mass spectrometry (QMS). Gene networks for ASD were constructed considering as input six of the eight associated loci due to the lack of information for the remaining two (**Table 2**). Gene networks were constructed considering three different parameters. Therefore, expansion parameters include confidence threshold (0.8), a maximum number of 30 nodes per expansion step, and a query depth of 1 (only genes directly linked to the query genes are shown). Network expansion algorithm was settled in order to obtain those strongest interactors for any query gene, without prioritizing common neighbor’s links. Moreover, enriched term analyses (KEGG, GO biological function, and GO molecular function) were considered for each gene network constructed with the corresponding p-values. Gene-network representation displays the most significant KEGG pathways according to their q-values after considering a FDR approach. Node sizes scales to emphasize gene importance in the whole network while participating nodes for each KEGG pathway are marked in black.

**Table 2:** FunCoup interactors detected using PASCAL associated genes as input. Some PASCAL- associated genes have not been detected by FunCoup (bold font). Those genes for which one or several interactors were found are shown in the table. Last column indicates the type of functional coupling among genes used to construct the network. Moreover, some PASCAL- associated genes and interactors were not detected by FUMA (underlined genes).

PASCAL analysis	Query genes for FunCoup	Interaction partners	Network
*XRN2*	*XRN2*	*DDX21,ILF2,CDKN2AIP,EXOSC8,NOC3L,ACIN1,ILF3,GNL3,KNRNPF,ADAR,LYAR,SNW1,HNRNPK,HNRNPH1,PARN,SUMO2,DHX15,NOP2,UPF1,ALYREF,RBM39,SYNCRIP,PTBP1,C14orf166,NONO,HNRNPUL1*	Complex and PPI
*NKX2-4*	*NKX2-2*	*OLIG2*	PPI
*KIZ*	*KCNN2*	*CALM1,CALM2,CALM3*	PPI
*KCNN2*			
*NKX2-2*			
***CRHR1-IT1***			
*C8orf74*			
***LOC644172***			

### Functional Annotation

GENE2FUNC, a core process of FUMA (*Functional Mapping and Annotation of Genome-Wide Association Studies*) (http://fuma.ctglab.nl/), was used to functionally annotate ASD genes and its interactors. Therefore, ASD input for FUMA comprises 36 genes (PASCAL associated genes plus strong interactors from FunCoup) (**Table 2**).

Different analyses performed by GENE2FUNC were employed, including a gene expression heatmap and an enrichment analysis of differentially expressed genes (DEG). Gene expression heatmap was constructed employing GTEx v7 (53 tissue types) and BrainSpan RNA-seq data. The average of normalized expression per label (zero means across samples) was displayed on the corresponding heatmaps. Expression values are TPM (Transcripts Per Million) for GTEx v7 and RPKM (Read Per Kilobase per Million) in the case of BrainSpan data set. Heatmaps display normalized expression value (zero mean normalization of log2 transformed expression), and darker red means higher relative expression of that gene in each label, compared to a darker blue color in the same label. DEG analysis was carried out creating differentially expressed genes for each expression data set. In order to define DEG sets, two-sided Student’s t-test were performed per gene per tissue against the remaining labels (tissue types or developmental stages). Those genes with a p-value < 0.05 after Bonferroni correction and a log fold change ≥ 0.58 are defined as DEG. The direction of expression was considered. The -log10 (p-value) refers to the probability of the hypergeometric test.

### Meta-Analysis of Differentially Expressed Genes in ASD Studies

dbMDEGA (https://dbmdega.shinyapps.io/dbMDEGA/) allows to conduct a meta-analysis using different ASD and brain control expression datasets to test which genes are differentially expressed (DEGs) between both groups. Specifically, this tool uses expression profiles from three human ASD brain gene expression datasets: GSE28475 (Chow et al., 2012), GSE28521 (Voineagu et al., 2011), and GSE38322 (Ginsberg et al., 2012). Thus, dbMDEGA contains about 17,741 human genes as meta-results for querying DEGs. PASCAL genes and FunCoup interactors (N = 36) were submitted in the Meta_summary panel. Meta-analysis p-values (random effects model), FDR (false discovery rate) for each gene, and heterogeneity measures (I^2^) were obtained.

## Results

### Gene-Based-Analysis

**Figure 1:**
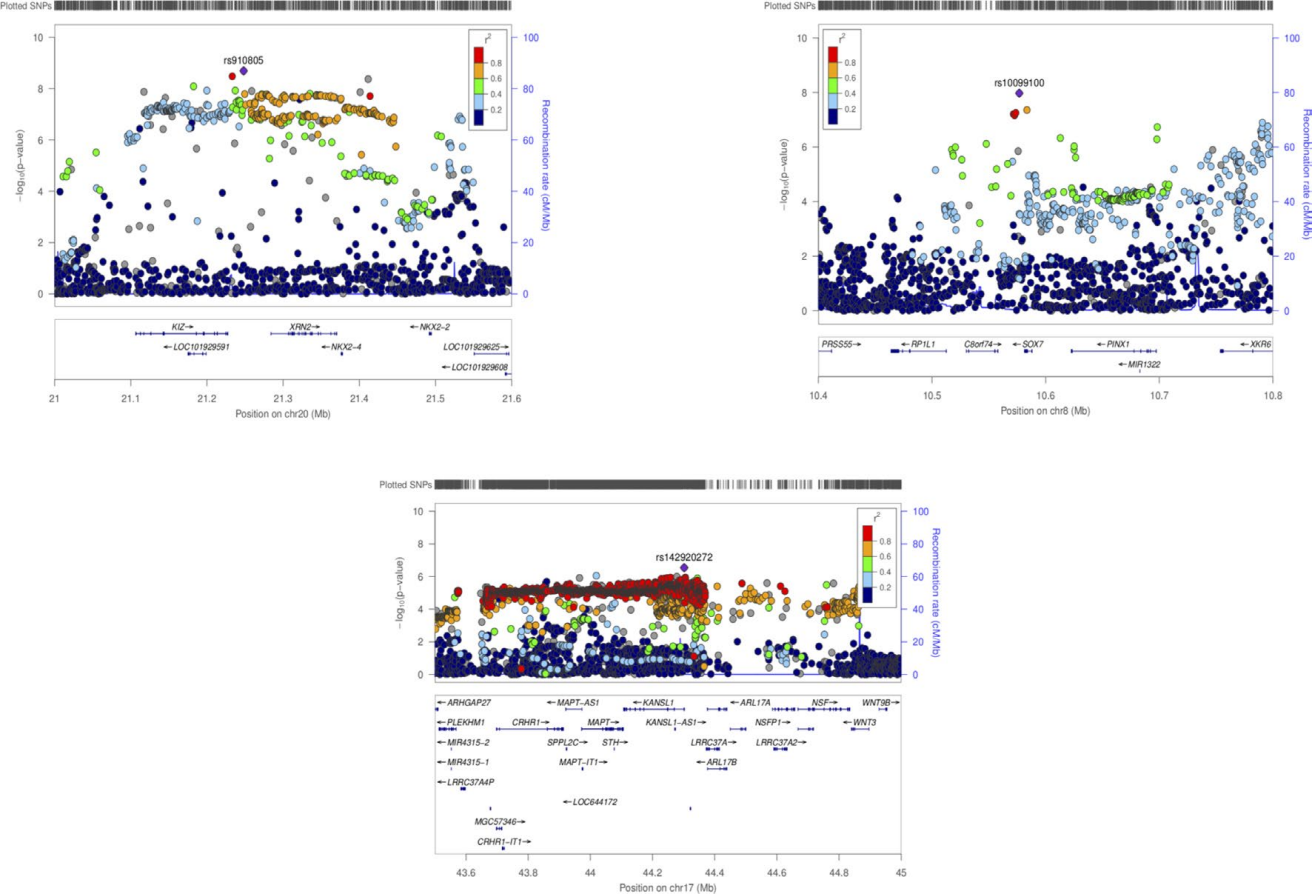
Regional association plots for ASD GWAS meta-analysis (chromosomes 20, 8 and 7). PASCAL has highlighted associations at a gene level for *NKX2*-2 and *NKX2-4* (chr20), *CRHR1-IT1*, *LOC644172* (chr17), and *C8orf74* (chr8) among other genes previously associated by MAGMA.

PASCAL analysis has revealed association of eight loci (p-value < 2.26 × 10^−6^) (**Table 3**). *NKX2-2* and *NKX2-4* (chromosome 20) were highlighted as associated by PASCAL in comparison with the results obtained by MAGMA (**Table 4**). Regional association plot around rs910805 shows three different r^2^ levels. PASCAL is able to detect *NKX2-2* and *NKX2-4* as associated, both genes located farther away from the lead SNP. However, SNPs within both loci are in moderate LD with the index SNP (**Figure 1**). In addition, PASCAL shows association of *CRHR1-IT1*, *LOC644172* (chromosome 17), and *C8orf74* (chromosome 8) (**Table 3** and **Figure 1**). It is worth to note that *CRHR1-IT1* is intronic with *CRHR1* (previously reported by MAGMA) (**Table 4**). However, the vast majority of loci associated by PASCAL were previously reported by MAGMA (**Table 4**).

**Table 3:** Top 20 results reported by PASCAL for ASD GWAS meta-analysis data. Genes represented in bold surpass Bonferroni threshold (p-value < 2.26 × 10^−^^6^). Columns show gene, chromosome, start and end positions for each gene, the number of SNPs included in the analysis for each gene, and their PASCAL p-values.

Gene	Chromosome	Start position	End position	Number of SNPs	PASCAL p-value
***XRN2***	chr20	21283941	21370463	271	3.53 × 10^−9^
***NKX2-4***	chr20	21376004	21378047	143	9.51 × 10^−9^
***PLK1S1***	chr20	21106623	21227258	287	4.69 × 10^−8^
***KCNN2***	chr5	113698015	113832197	540	3.89 × 10^−7^
***NKX2-2***	chr20	21491659	21494664	166	7.78 × 10^−7^
***CRHR1-IT1***	chr17	43716340	43723595	26	1.69 × 10^−6^
***C8orf74***	chr8	10530146	10558103	314	1.78 × 10^−6^
***LOC644172***	chr17	43677490	43679748	24	2.15 × 10^−6^
*LRRC37A*	chr17	44372496	44415160	48	2.64 × 10^−6^
*ARL17A*	chr17	44363861	44657088	64	2.82 × 10^−6^
*KANSL1-AS1*	chr17	44270938	44274089	46	2.84 × 10^−6^
*KANSL1*	chr17	44107281	44302740	153	3.07 × 10^−6^
*MAPT-IT1*	chr17	43973148	43976164	50	3.68 × 10^−6^
*SOX7*	chr8	10581277	10697299	752	4.84 × 10^−6^
*MAPT*	chr17	43971747	44105699	100	4.86 × 10^−6^
*CRHR1*	chr17	43697709	43913194	275	5.45 × 10^−6^
*MAPT-AS1*	chr17	43920721	43972879	224	6.14 × 10^−6^
*STH*	chr17	44076615	44077060	37	6.27 × 10^−6^
*SPPL2C*	chr17	43922255	43924438	193	6.57 × 10^−6^
*PINX1*	chr8	10622883	10697299	684	7.78 × 10^−6^

**Table 4:** Significant genes reported by PASCAL and MAGMA algorithms. All significant genes reported in our study and Grove et al. publication are shown. Columns show chromosome, start and end positions for each gene, p-value reported by PASCAL or MAGMA, and the corresponding tag SNP for each gene.

Gene	Chromosome	Start position	End position	PASCAL/MAGMA p-value	Tag SNP
**Results from PASCAL gene based analysis**					
*XRN2*	20	21283941	21370463	3.53 × 10^−9^	rs 910805
*NKX2-4*	20	21376004	21378047	9.51 × 10^−9^	rs 910805
*PLK1S1*	20	21106623	21227258	4.69 × 10^−8^	rs 910805
*KCNN2*	5	113698015	113832197	3.89 × 10^−7^	rs 13188074
*NKX2-2*	20	21491659	21494664	7.78 × 10^−7^	rs 910805
*CRHR1-IT1*	17	43716340	43723595	1.69 × 10^−6^	rs 142920272
*C8orf74*	8	10530146	10558103	1.78 × 10^−6^	rs 10099100
*LOC644172*	17	43677490	43679748	2.15 × 10^−6^	rs 142920272
**Results from MAGMA gene based analysis (** Grove et al, 2019 **)**					
*XRN2*	20	21283942	21370463	9.69 × 10^−10^	rs 910805
*KCNN2*	5	113698016	113832197	1.02 × 10^−9^	rs 13188074
*PLK1S1*	20	21106624	21227260	5.17 × 10^−9^	rs 910805
*MACROD2*	20	13976146	16033842	1.40 × 10^−7^	rs 71190156
*WNT3*	17	44841686	44896082	4.03 × 10^−7^	rs 142920272
*MAPT*	17	43971748	44105700	5.01 × 10^−7^	rs 142920272
*MFHAS1*	8	8641999	8751131	5.58 × 10^−7^	rs 11249905
*XKR6*	8	10753654	11058875	8.01 × 10^−7^	rs 10099100
*MSRA*	8	9911830	10286401	9.15 × 10^−6^	rs 10099100
*CRHR1*	17	43697710	43913194	1.07 × 10^−6^	rs 142920272
*SOX7*	8	10581278	10588022	1.24 × 10^−6^	rs 10099100
*NTM*	11	131240371	132206716	1.32 × 10^−6^	rs 549507
*MMP12*	11	102733464	102745764	2.28 × 10^−6^	rs 102751102
*BLK*	8	11351521	11422108	2.45 × 10^−6^	rs 2736342

### Gene-Network Analysis

**Figure 2:**
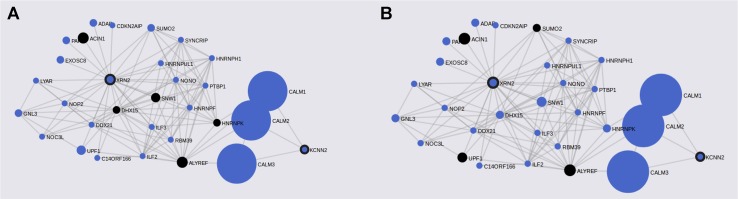
ASD gene networks constructed with PASCAL associated genes and its FunCoup interactors. Node sizes scale to emphasize gene importance in the whole network, while participating nodes for each KEGG pathway are marked in black: **(A)** cAMP signaling pathway; **(B)** RNA transport.

FunCoup has detected several interactors for PASCAL associated loci (**Table 2**). Gene network for ASD genes was constructed after including 36 genes (30 subnetwork genes plus 6 query genes) and considering 120 links between them (**Table 2**). FunCoup was not able to detect interactors for the remaining ASD-associated loci. Enrichment analysis for KEGG and GO terms has shown association of different biological processes as spliceosome (q-value = 3 × 10^−4^), RNA transport (q-value = 2.8 × 10^−3^), and nucleic acid binding (q-value = 1.09 × 10^−14^) (**Table 5** and **Figure 2**).

**Table 5:** Enriched terms for query ASD genes and its interactors (subnetwork genes) according to FunCoup.

Enriched terms		Genes	q value
KEGG metabolic	Spliceosome	*DH15, HNRNPK, ACIN1, ALYREF, SNW1*	3 × 10^−4^
	RNA transport	*SUMO2, ACIN1, ALYREF, UPF1*	2.8 × 10^−3^
GO molecular function	Nucleic acid binding	*DDX21, ILF2, CDKN2AIP, EXOSC8, NOC3L, ACIN1, ILF3, GNL3, KNRNPF, ADAR, LYAR, SNW1, HNRNPK, HNRNPH1,PARN, SUMO2, DHX15, NOP2, UPF1,ALYREF, RBM39, SYNCRIP, PTBP1, C14orf166, NONO,HNRNPUL1, XRN2, NKX2-4, NKX2-2*	1.09 × 10^−14^
	Heterocyclid compound binding	*DDX21,ILF2,CDKN2AIP,EXOSC8,NOC3L,ACIN1,ILF3,GNL3,KNRNPF,ADAR,LYAR,SNW1,HNRNPK,HNRNPH1,PARN,SUMO2,DHX15,NOP2,UPF1,ALYREF,RBM39,SYNCRIP,PTBP1,C14orf166,NONO,HNRNPUL1,XRN2,NKX2-4,NKX2-2*	3.88 × 10^−10^
	Organic cyclid compound binding	*DDX21, ILF2, CDKN2AIP, EXOSC8, NOC3L, ACIN1, ILF3, GNL3, KNRNPF, ADAR, LYAR, SNW1, HNRNPK, HNRNPH1, PARN, SUMO2, DHX15, NOP2, UPF1, ALYREF, RBM39, SYNCRIP, PTBP1, C14orf166,NONO, HNRNPUL1, XRN2, NKX2-4, NKX2-2*	3.88 × 10^−10^
	Protein binding	*DDX21, ILF2, CDKN2AIP, EXOSC8, ACIN1, ILF3, GNL3, KNRNPF, ADAR, LYAR, SNW1, HNRNPK, HNRNPH1, PARN, SUMO2, DHX15, NOP2, UPF1, ALYREF, RBM39, SYNCRIP, PTBP1,C14orf166, NONO, HNRNPUL1, XRN2, KCNN2, NKX2-2, KIZ, C8orf74*	9.94 × 10^−4^
	Binding	*DDX21,ILF2,CDKN2AIP, EXOSC8, ACIN1,ILF3,GNL3, KNRNPF,ADAR,LYAR, SNW1,HNRNPK, HNRNPH1,PARN,SUMO2, DHX15,NOP2, UPF1, ALYREF, RBM39, SYNCRIP, PTBP1,C14orf166,NONO, HNRNPUL1, XRN2,KCNN2, NKX2-2,KIZ, C8orf74, NKX2-4, NOC3L*	2.81 × 10^−2^
	Chromatin binding	*NKX2-2, NONO, NOC3L, UPF1,*	1.03 × 10^−1^
	Transcription factor binding	*NKX2-2, HNRNPF, SUMO2, SNW1*	1.03 × 10^−1^
	Enzyme binding	*KIZ, SUMO2,C14orf166, PARN, ACIN1, HNRN,UL1, SNW1*	2.54 × 10^−1^
	Hydrolase activity, acting on acid anhydrides	*DHX15, DDX21,ACIN1,UPF1*	3.34 × 10^−1^
	Identical protein binding	*KCNN2, NONO, EXOSC8, C14orf166,OLIG2*	4.21 × 10^−1^
	hydrolase activity	*XRN2, DHX15,ADAR, DDX21,PARN,ACIN1,UPF1*	6.03 × 10^−1^

### Functional Annotation

#### Gene Expression Heatmaps and Differential Expression Analysis

**Figure 3:**
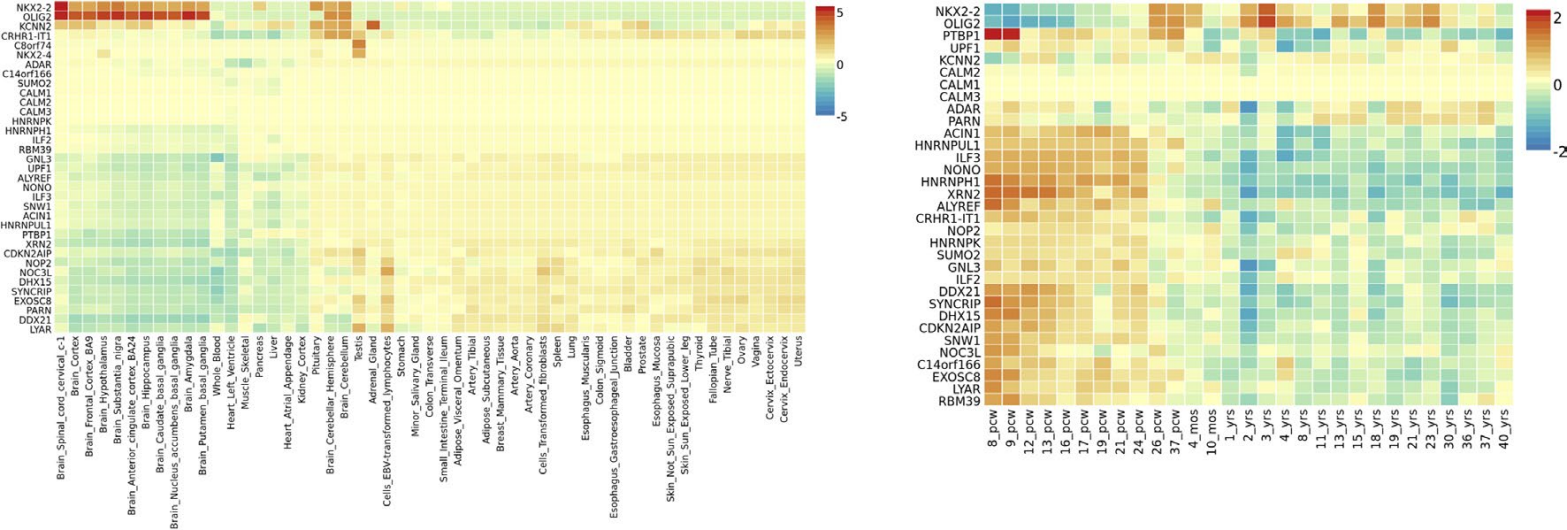
ASD gene expression heatmaps constructed with GTEX v7 (53 tissues) (left) and BrainSpan 29 different ages of brain samples data (right). Genes and tissues are ordered by clusters for the GTEX heatmap. In the case of BrainSpan heatmap, genes are ordered by expression clusters, and neurodevelopmental stages are chronologically classified from prenatal to postnatal stages.

**Figure 4:**
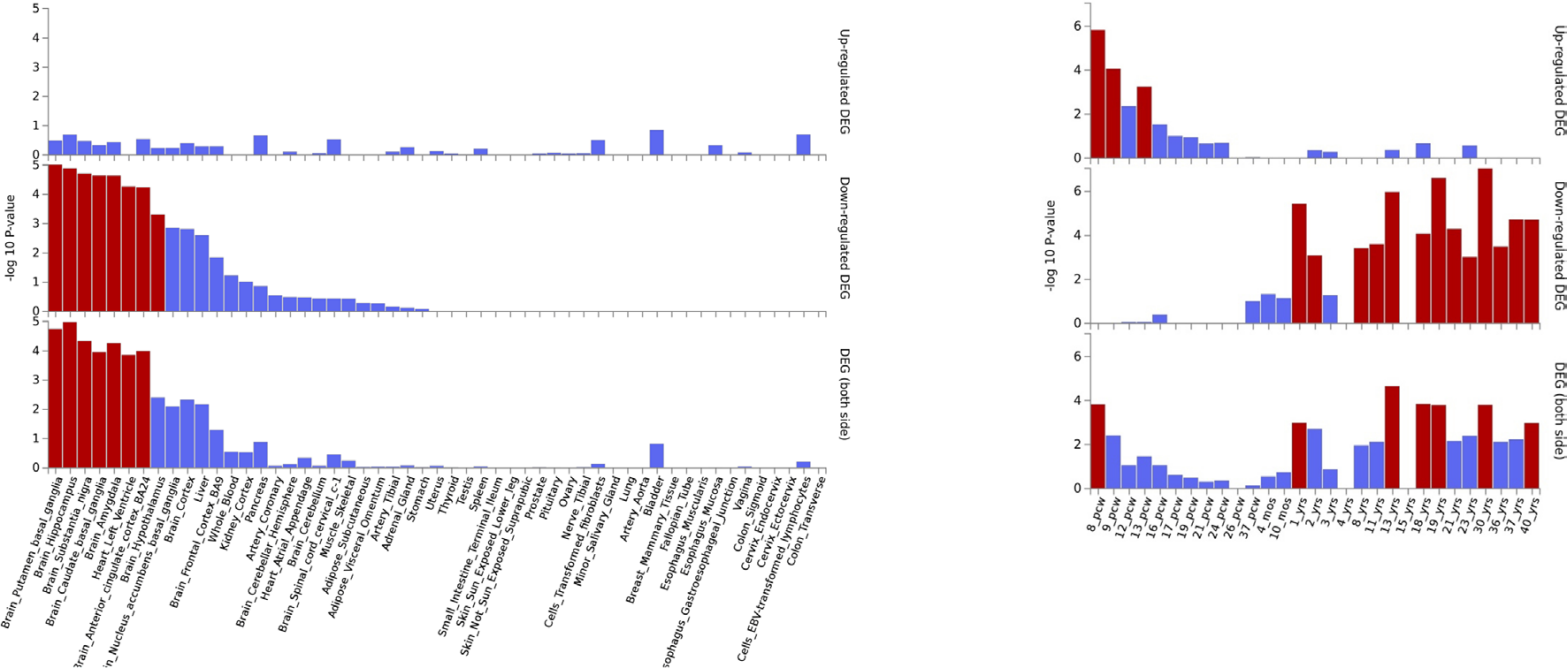
ASD DEG plots constructed with GTEX v7 (53 tissues) (left) and BrainSpan 29 different ages of brain samples data (right). Significantly enriched DEG sets (Pbon < 0.05) are highlighted in red.

Gene expression heatmap based on GTEX v7 RNA-seq data for ASD-associated genes together with FunCoup interactors (**Table 2**) has revealed interesting results. Specifically, three genes (*NKX2-2*, *OLIG-2*, and *KCNN2*) display increased expression levels in brain (different regions) in comparison with the remaining tissues included in the analysis. Moreover, another gene set, *XRN2* plus some interactors, display the opposite trend showing lower relative expression levels on brain tissues (**Figure 3**). Interestingly, gene expression heatmap employing BrainSpan data has revealed that the vast majority of genes included on the downregulated gene-set have shown a higher relative expression in prenatal stages (early, early-mid, and late-mid). Moreover, the same trend was also observed for *NKX2-2* and *OLIG2*. Therefore, a lower relative expression level for these genes was shown across prenatal stages in comparison with those levels found in adult brain tissues (**Figure 3**). Thus, there is a clear division in terms of expression levels for those sets of genes expressed across different pcw (post-conception weeks) *versus* those expressed across the adult lifespan. DEG analysis with GTEx data (adult brain) has shown significantly enriched DEG sets across distinct brain tissues. Moreover, DEG study performed with BrainSpan data has confirmed a significant association of gene sets during early prenatal stages (up-regulation) *versus* young adulthood (down-regulation). Specifically, up-regulation occurs around 8, 9, and 13 pcw, and down-regulation takes place across different postnatal ages (**Figure 4**).

### Meta-Analysis of Differentially Expressed Genes in ASD Studies

Expression meta-analysis for PASCAL associated genes has shown differentially expressed genes between ASD brain individuals and controls (*XRN2*, p-value = 0.02; *KCNN2*, p-value = 0.01; *C8orf74*; p-value = 0.03). Moreover, some of the *XRN2* interactors have also shown significant p-values when dbDMEGA analysis was performed (*XRN2*, *CDKN2AIP*, *ILF3*, *GNL3 ADAR*, *LYAR*, *SNW1*, *RBM39*, *SYNCRIP*, and *PTBP1*) (**Table 6**).

**Table 6:** PASCAL associated genes and interactors. p-values from PASCAL and dbMDEGA meta-analysis are shown for each gene. p-values from dbMDEGA were computed using the p-value method. Heterogeneity values (I^2^) are also shown. (NA = the gene is not in dbMDEGA).

Gene	p-val PASCAL	p-val dbMDEGA	FDR dbMDEGA	Heterogeneity
*XRN2*	3.52863316 × 10^−9^	0.02	0.12	0%
*NKX2-4*	9.5086814 × 10^−9^	NA	NA	NA
*PLK1S1*	4.68346333 × 10^−8^	NA	NA	NA
*KCNN2*	3.88782618 × 10^−7^	0.01	0.11	0%
*NKX2-2*	7.7773789 × 10^−7^	0.23	0.37	0%
*CRHR1-IT1*	1.6858736 × 10^−6^	NA	NA	NA
*C8orf74*	1.77348539 × 10^−6^	0.03	0.16	43%
*LOC644172*	2.15004243 × 10^−6^	NA	NA	NA
*DDX21*	8.86663103 × 10^−1^	0.3	0.41	69%
*ILF2*	5.52756293 × 10^−1^	0.38	0.45	28%
*CDKN2AIP*	8.21802658 × 10^−1^	0.04	0.17	0%
*EXOSC8*	9.41352461 × 10^−2^	0.1	0.26	64%
*NOC3L*	3.66793335 × 10^−1^	0.35	0.44	0%
*ACIN1*	9.65129025 × 10^−1^	0.15	0.3	48%
*ILF3*	1.0116456 × 10^−1^	0.01	0.1	66%
*GNL3*	2.39022069 × 10^−3^	0.01	0.07	67%
*KNRNPF*	NA	NA	NA	NA
*ADAR*	1.70702835 × 10^−2^	0.01	0.1	72%
*LYAR*	2.30069599 × 10^−1^	0.03	0.15	0%
*SNW1*	1.0892706 × 10^−1^	0.04	0.18	74%
*HNRNPK*	3.13685174 × 10^−1^	NA	NA	NA
*HNRNPH1*	6.07006486 × 10^−1^	NA	NA	NA
*PARN*	9.01671731 × 10^−1^	0	0.03	79%
*SUMO2*	8.85347296 × 10^−1^	0.27	0.39	72%
*DHX15*	2.93250866 × 10^−1^	0.12	0.38	0%
*NOP2*	7.85747107 × 10^−1^	NA	NA	NA
*UPF1*	2.08668839 × 10^−1^	0.12	0.27	14%
*ALYREF*	2.00036414 × 10^−1^	NA	NA	NA
*RBM39*	2.90163509 × 10^−1^	0.06	0.2	18%
*SYNCRIP*	2.62087002 × 10^−1^	0.01	0.08	0%
*PTBP1*	9.27312631 × 10^−2^	0.02	0.13	0%
*C14orf166*	2.91369259 × 10^−1^	0.36	0.44	58%
*NONO*	NA	0.18	0.33	32%
*HNRNPUL1*	4.2805356 × 10^−1^	NA	NA	NA
*CALM1*	3.30876236 × 10^−1^	0.4	0.46	27%
*CALM2*	9.81305815 × 10^−1^	0.36	0.44	0%
*CALM3*	7.91761032 × 10^−1^	0.5	0.5	0%
*OLIG2*	4.52957535 × 10^−1^	0.4	0.46	0%

## Discussion

The latest ASD GWAS meta-analysis has reported the association of individual SNPs across chromosome 20 (rs910805), 5 (rs13188074), 17 (rs142920272), and 8 (rs10099100), among others. In the same study, different genes located near to the top-ranked SNPs as *XRN2*, *KCNN2*, and *KIZ* (or *PLK1S1*) have been associated by MAGMA (Grove et al., 2019). PASCAL analysis has shown additional associated genes: *NKX2-4*, *NKX2-2*, *CRHR1-IT1*, *C8orf74*, and *LOC644172*. However, these findings should not be considered as completely new. Thus, it is worth noting that lead SNPs located on chromosome 20 and 8 were associated with ASD when MTAG (*multi-trait analysis of GWAS*) was done with genetically correlated phenotypes (schizophrenia and educational attainment) but not by MAGMA itself (Turley et al., 2018; Grove et al., 2019). In fact, this is why *NKX2-4* and *NKX2-2* were reported as “possibly associated” genes when MTAG was carried out (Grove et al., 2019). However, we found that PASCAL is able to rescue these associations when its own statistical approach is applied. It is also worth to note that *CRHR1-IT1* partially overlap with *CRHR1* even if it encodes a different transcript (previously not reported by MAGMA). Thus, *CHR1-IT1* is reported as associated by PASCAL due to the inclusion of the “gene-name” in the list used as input (present in PASCAL and not in MAGMA). c*8orf74* could also be a controversial “new” finding because the SNPs located within the gene harbor more significant p-values but are located near to *XKR6* (identified in Grove et al, 2019). Even so, it should be considered that PASCAL has helped to unveil the association of additional genes near to those genes reported by MAGMA. Both GBA tools, MAGMA and PASCAL, work in a very similar way: i) they are able to employ summary statistics as input instead of genotypes, ii) gene scores are calculated combining the results for all SNPs located across the gene, and iii) LD correction is made by external information from the 1000 Genomes European panel (de Leeuw et al., 2015; Lamparter et al., 2016). However, the construction of the correlation matrix is slightly different between both methods. This could explain those small differences found between the associated genes reported by both methods. p-values associated to each gene in PASCAL were in general less significant than those reported by MAGMA, even when identical genes were reported by both algorithms. Moreover, the number of genes that remain significant after Bonferroni correction was lower in PASCAL. All these results taken together could indicate that PASCAL is more conservative than MAGMA. In addition, we propose that PASCAL could be used as a complementary GBA approach because it has helped to highlight additional genes located in the same LD region than those reported by MAGMA.

Regarding the insight into the biological role of ASD-associated genes, we would like to focus on the following loci: *NKX2-2*, *NKX2-4*, *CRHR1-IT1*, *LOC644172*, and *c8orf74*. *NKX2-2* and *NKX2-4* are members of the homeobox-transcription factor family. *NKX2-2* encodes a transcription factor involved in the morphogenesis of the central nervous system, and it is essential during the differentiation of neural populations located on the hindbrain and spinal cord during early development (Briscoe and Ericson, 1999). *NKX2-4* (*Homeobox Protein Nkx-2.4*) has a key role in brain development, and its downregulation in the developing forebrain promotes proliferation and inhibits differentiation of neural progenitors which results in impaired neurogenesis (Shen et al., 2018). Gene expression heatmaps (GTEX and BrainSpan data) have revealed two expression clusters in which *NKX2-2* is involved. Genes within the first cluster have shown downregulation across early prenatal stages and upregulation during postnatal stages, and genes within the second cluster have shown the opposite trend. *NKX2-2* and its interactor *OLIG-2* (*oligodendrocyte transcription factor 2*) are included in the first cluster that displays a lower normalized expression during prenatal stages in comparison with postnatal stages. This seems to point to the existence of a different genetic regulation for *NKX2-2* during brain development in comparison with the adult brain. Thus, it was reported that *NKX2-2* is initially expressed in differentiating oligodendrocyte precursor cells (OPCs) and then is downregulated. However, it was demonstrated that its expression could also be upregulated again during later neurodevelopmental stages in order to maintain myelin structures (Cai et al., 2010). This is particularly interesting due to previous reports that have linked aberrant expression of genes across the oligodendrocyte lineage with ASD pathology (Li et al., 2014; Zeidán-Chuliá et al., 2016). In addition, enriched terms for *NKX2-2* and *OLIG-2* were protein, chromatin, and TF binding, all of them biological processes involved in ASD pathogenesis. However, it should be noted that both genes did not show differences in expression between ASD brain tissue and controls when DEG was carried out. These results indicate the need of subsequent functional studies to further characterize their biological role.

In addition, a second ASD expression cluster (BrainSpan heatmap) includes *XRN2*, *CRHR1-IT1*, and their multiple interactors. First of all, it should be remarked that FunCoup calculates many more interactors for *XRN2* than for the remaining genes. This could entail a bias for subsequent analysis but also underlines the relevance of this gene due to its wide PPI network. Thus, it is worth to note that many *XRN2* and some of its interactors (*CDKN2AIP*, *ILF3*, *GNL3 ADAR*, *LYAR*, *SNW1*, *RBM39*, *SYNCRIP*, and *PTBP1*) have shown significant p-values when dbMDEGA meta-analysis was performed. This fact demonstrates that these genes are differentially expressed between ASD brains and controls and therefore their potential relevance in ASD pathogenesis (Voineagu et al., 2011; Chow et al., 2012; Ginsberg et al., 2012). In addition, some of the enriched terms for *XNR2* and their interactors were spliceosome, RNA transport, and nucleic acid binding, all of them essential biological functions with a role during early development. Thus, it was demonstrated that *XRN2* might serve a general role in triggering the termination of transcription through the degradation of the 3′ UTR (Eaton et al., 2018). *CRHR1-IT1* codifies for a long intergenic non-protein coding RNA which was recently associated with susceptibility to antisocial behavior (Day et al., 2018). *CRHR1-IT1* shares part of its sequence with *CRHR1* which encodes the corticotropin-releasing hormone receptor 1 (Grove et al., 2019). *CRHR1* is a main component of the hypothalamic-pituitary-adrenal pathway, and it has been repeatedly associated with response to stress-related psychopathology (Binder and Nemeroff, 2010). The function of *CRHR1-IT1* is not well characterized, but it might play a role in the regulation of *CRHR1* expression. Thus, *CRHR1* and *CRHR1-IT1* could be involved in the modulation of behavior and cognition, both core features of ASD (Smith et al., 2016; Grimm et al., 2017; Day et al., 2018; Zhong et al., 2018). However, the meta-analysis of DEG in ASD brain *vs* controls has not revealed association for *CRHR1* nor *CRHR1-IT1*.

Finally, *C8orf74* and *LOC644172* were also associated after PASCAL analysis. *LOC644172* is located upstream of *CRHR1* and *CRHR1-IT1*, but it was not recognized by FunCoup nor FUMA. *C8orf74* is part of a reading frame that has the ability to be translated, but it is not yet characterized. Thus, it seems that both loci are not included in functional and genetic databases due to their poor biological characterization. However, the meta-analysis of expression profiles revealed that *C8orf74* was differentially expressed between ASD brain tissues and controls, which indicates that *C8orf74* needs further functional characterization.

## Conclusions

PASCAL algorithm was used to carry out a novel GBA of ASD, employing summary statistics from the latest GWAS meta-analysis. This study has identified several gene associations even though most of them were previously reported by MAGMA. However, PASCAL has been useful to define the association of other genes located in the same LD region than those found by MAGMA. These results indicate that PASCAL should be considered as a complementary GBA approach when it is necessary to further mine GWAS results.

The second part of this study has been focused on the biological characterization of the ASD-associated genes by PASCAL. Thus, gene-network and functional annotation approaches including gene expression heatmaps and DEG were carried out. This has helped to understand the genetic findings into a biological context and to select the most suitable ASD susceptibility genes as candidates for functional biological studies.

## Data Availability

All data generated during this study are included in this published article and its supplementary information files. Summary statistics for ASD GWAS are publicly available at https://www.med.unc.edu/pgc/results-and-downloads.

## Ethical Statements

The GWAS data employed in this study are publicly available at https://www.med.unc.edu/pgc. These genetic data have been employed in several published studies and have been approved by the corresponding ethics committees.

## Author Contributions

AA-G, CR-F and MCC have performed the analyses. AA-G wrote the paper. AC and CR-F critically revised the work and approved the final content. AA-G, CR-F, MCC, and AC participated in the design and coordination of this study.

## Funding

AA-G was supported by Fundación María José Jove. CR-F was supported by a contract from the ISCIII and FEDER.

## Conflict of Interest Statement

The authors declare that the research was conducted in the absence of any commercial or financial relationships that could be construed as a potential conflict of interest.

## Abbreviations

ASD, autism spectrum disorder; GWAS, genome-wide association study; SNP, single-nucleotide polymorphism; GBA, gene-based analysis; LD, linkage disequilibrium; PASCAL, Pathway scoring algorithm; KEGG, Kyoto Encyclopedia of Genes and Genomes; GO, gene ontology; DEA, differential expression analysis; GTEx, genotype-tissue expression; NDD, neurodevelopmental disorder; CNV, copy number variation; PGC, psychiatric genomic consortium; VEGAS, versatile gene-based association study; iPSYCH, Lundbeck Foundation Initiative for Integrative Psychiatric Research; QC, quality control; OR, odd ratio; PIN, protein interaction; MEX, Mrna co-expression; PEX, protein co-expression; GIN, genetic interaction profile similarity; TFB, shared transcription factor binding; MIR, co-miRNA regulation by shared miRNA targeting; SCL, subcellular co-localization; DOM, domain interactions; PHP, phylogenetic profile similarity; QMS, quantitative mass spectrometry; FDR, false discovery rate; FUMA, functional mapping and annotation of genome-wide association studies; DEG, differentially expressed genes; TPM, transcripts per million; RPKM, read per kilobase per million; MTAG, multi-trait analysis of GWAS.
